# Rare Challenges in Diagnosing Cushing’s Syndrome and Primary Aldosteronism: A Case Report of a Female With a Negative Workup

**DOI:** 10.7759/cureus.42589

**Published:** 2023-07-28

**Authors:** Asiya Jatoi, Yasser Haider-Badenhorst

**Affiliations:** 1 Endocrinology, Christie Clinic, Urbana, USA; 2 Medicine, Ziauddin University, Karachi, PAK; 3 Clinical Sciences, University of Illinois at Urbana-Champaign, Urbana, USA

**Keywords:** cushing’s syndrome, adrenal cortical adenoma, adrenal veinous sampling, glucocorticoid-remediable aldosteronism, aldosteronism

## Abstract

Cushing’s syndrome with concurrent primary aldosteronism (PA) is a rare presentation, and establishing an early diagnosis is imperative to preventing morbidity and long-term sequelae. The diagnosis is established by sequential lab work, showing an elevated cortisol and aldosterone level.

Taking the above into consideration, it is evident that repeatedly negative results on all three tests can present an extremely challenging case. In this report, we discuss a female who presented with an adrenal incidentaloma and features suggestive of primary hyperaldosteronism as well as Cushing’s syndrome but no elevations in serum, urine, or salivary cortisol.

In this study, we present a 37-year-old female with resistant hypertension and tachycardia. She had several features suggestive of Cushing’s syndrome including resistant hypertension, proximal muscle weakness, weight gain, easy bruising, hair loss, and a history of tachycardia and chest pain. Examination revealed an obese female with thin silvery abdominal striae. The patient’s labs revealed normal serum cortisol, urine-free cortisol (UFC), late-night salivary cortisol, and a normal dexamethasone suppression test. An abdominal computed tomography (CT) scan revealed a right adrenal mass measuring 2.1 x 1.5 x 2.5 cm. Due to a high index of suspicion, adrenal venous sampling was performed, which revealed high levels of cortisol and aldosterone in the right vein, confirming the diagnosis. The patient subsequently underwent a right adrenalectomy. She developed hypotension post-op, leading to the diagnosis of glucocorticoid-remediable aldosteronism.

## Introduction

Primary aldosteronism (PA) is the excess production of aldosterone by the adrenal glands, despite a low serum renin level. The presentation of hyperaldosteronism can be vague and include symptoms such as muscle weakness, fatigue, headaches, numbness, and cramps. More specific findings include resistant hypertension, low serum potassium, and metabolic alkalosis. The etiologies are variable and can include an adrenal adenoma (Conn syndrome) or bilateral adrenal hyperplasia [1].

Cushing’s syndrome is also caused by excess hormone secretion by the adrenal glands. The etiologies include a primary adrenal adenoma, hyperplasia, carcinoma, or exogenous corticosteroid use. It can also be caused by an adrenocorticotropic hormone (ACTH)-secreting pituitary adenoma or as a result of paraneoplastic ACTH secretion. The clinical presentation is highly variable and leads to difficulties in establishing a diagnosis.

The concurrent existence of primary hyperaldosteronism and Cushing’s syndrome creates additional hindrances in diagnosis, yet further obscured in a patient with a repeatedly negative workup for both conditions.

## Case presentation

**Figure 1:**
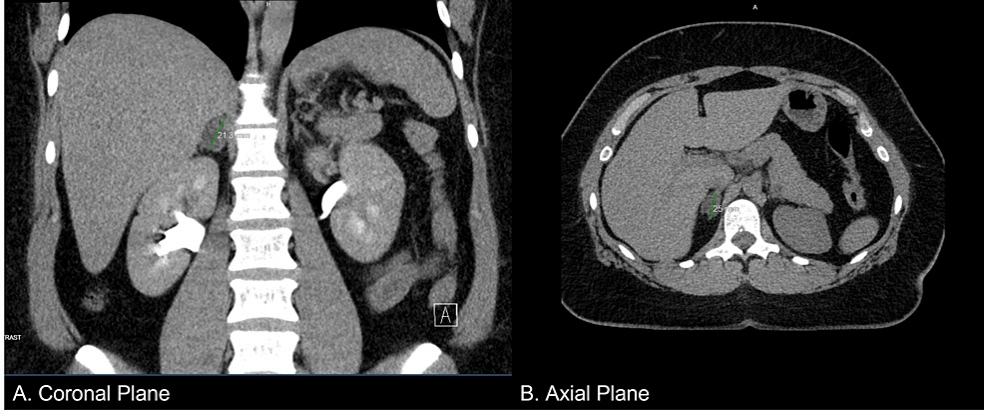
Abdominal CT scan showing a nodule in the right adrenal gland measuring 2.1 x 1.5 x 2.5 cm

A 37-year-old female presented to her primary care physician with complaints of proximal muscle weakness, tachycardia, and chest pain. Repeated blood pressure readings revealed that she was hypertensive, and she was started on amlodipine and benazepril, which elevated her blood pressure further. A computed tomography (CT) scan (Figure 1) of the abdomen was performed due to resistant hypertension, which revealed an adrenal incidentaloma (right adrenal gland measuring 2.1 x 1.5 x 2.5 cm). Precontract density was 5 Hounsfield units, and a 15-minute delayed washout showed 11 Hounsfield units for a 72% washout. She was thus referred to endocrinology.

She presented to the endocrinology clinic on March 12, 2021. A thorough physical examination was performed, which revealed a well-appearing obese female (BMI of 38.86 kg/m^2^) with no acute distress. Her blood pressure was 144/108 mmHg, her pulse was 95, and she was afebrile. Thin silvery striations were present on the abdomen, and alopecia was present on the crown. A review of all other systems was unremarkable. A detailed family history revealed early-onset hypertension in her brother (age: 35 years) and her mother (age: 30 years). Personal history included elevated anxiety, weight gain, headaches (frontal band distribution), increased thirst, easy bruising as well as delayed clearance of bruises, and proximal muscle weakness presenting as difficulty in climbing stairs and inability to lift heavy objects. She reported no change in menstrual cycles. There was no history of exogenous corticosteroid use.

Serum biochemistries were sent (Table 1), which showed normal levels of thyroid stimulating hormone (TSH), creatinine, liver function tests, and serum electrolytes. However, mildly elevated aldosterone (23 ng/dl), mild hypokalemia (3.3 mEq/L), and suppressed ACTH and dehydroepiandrosterone (DHEA) sulfate were discovered. The aldosterone to renin ratio was also elevated at 59.9 on spironolactone and was 71.4 three months later when spironolactone was discontinued. These findings lead to a preliminary diagnosis of primary hyperaldosteronism.

**Table 1:** Patient serum biochemistries BUN: Blood urea nitrogen; AST: Aspartate transaminase; ALT: Alanine transaminase.

Test	Result
Calcium	9.1 mmol/L
Sodium	137 mmol/L
Potassium	4.1 mmol/L
Chloride	106 mmol/L
CO_2_	27
BUN	15 mmol/L
Glucose	95 mmol/L
Creatinine	1.1 μmol/L
AST	24 U/L
ALT	20 U/L
Albumin	4.4 g/L
Total protein	7.0 g/L
Total bilirubin	0.4 μmol/L
Alkaline phosphatase	40 U/L
Renin	0.44

A workup for elevated cortisol was also performed as the patient was phenotypically Cushingoid, and the following biochemistries were sent sequentially: serum cortisol, 24-hour urine-free cortisol (UFC), salivary cortisol, and a low-dose dexamethasone suppression test (Table 2). The bloodwork was hence nonconfirmatory.

**Table 2:** Patient follow-up bloodwork

Endocrine workup	
Serum cortisol	4.5 mcg/dL
Urine-free cortisol	1.57 g/24 h
Salivary cortisol	<0.03 μg/dL
Dexamethasone suppression test	1.5 mcg/dL
Aldosterone	<4.0

Despite a repeatedly negative workup for Cushing's syndrome, adrenal venous sampling was performed due to a high index of suspicion. The results revealed an inferior vena cava (IVC) cortisol of 20, left adrenal venous (LAV) cortisol of 81, and right adrenal vein (RAV) cortisol of 1280. The results of the IVC aldosterone were 24, LAV aldosterone was 660 and RAV aldosterone was 1500. The elevated levels of cortisol in the RAV were in complete contradiction to the aforementioned workup. A diagnosis of Cushing’s syndrome and concurrent PA was determined.

Adrenal veinous sampling was instrumental in establishing the diagnosis but was equivocal and did not lateralize aldosterone and cortisol excess. However, the amount of aldosterone and cortisol were both significantly higher on the right side. After a panel discussion with doctors from several disciplines, a laparoscopic adrenalectomy was planned. The procedure was successful, and the patient was initially showing clinical improvement. The specimen was sent for pathological evaluation and revealed an adrenal cortical adenoma.

After initial improvement, the patient developed hypotension, which was likely due to adrenal insufficiency. The patient was supplemented with 1-mg dexamethasone tablets, which stabilized her condition, and a diagnosis of glucocorticoid-remediable-aldosteronism was made.

Based on a strong family history of early onset-resistant hypertension, a genetic component was suspected. Several genes associated with PA with autosomal dominant inheritance have been identified [2], such as CYP11B2, CLCN2, KCNJ5, CACNA1D, and CACNA1H. The patient was offered genetic testing but was unable to follow through due to financial reasons.

## Discussion

This patient presented as an extremely rare example of PA and Cushing’s syndrome, with negative serum cortisol, 24-hour UFC, late-night salivary cortisol, and a dexamethasone suppression test. Despite repeatedly negative lab results, the patient presented with a markedly elevated cortisol on adrenal venous sampling. In our literature search, we found an instance of a patient with several negative UFCs [3]; however, to the best of our knowledge, there have been no reported instances of a completely negative workup in a patient who is positive for Cushing’s syndrome. In fact, in the practice guidelines published by the Journal of Clinical Endocrinology & Metabolism [4], it is recommended that patients with a suspected diagnosis of Cushing’s syndrome or an adrenal incidentaloma and two concordant negative test results need not undergo further investigations.

One proposed mechanism for the misleading workup could be assay interference. Interference occurs when a substance or process falsely alters an assay result [5]. This can lead to incorrect diagnosis and subsequent treatment and poses a threat to the patient. Another suggested mechanism causing false negative test results could be the hook effect [6]. The hook effect is described as a phenomenon that leads to falsely low results due to the presence of excessive analyte.

In a study by Friedman et al. [7], it was noted that patients with “episodic Cushing’s syndrome” or those with mild symptoms had a negative workup. The study recommended serial monitoring for the disease. The interesting fact is that our patient had several features suggestive of active Cushing’s syndrome, and the hypotension seen postoperatively was a testament to the fact that there was in fact a cortisol excess, which led to adrenal insufficiency. In light of the above, a consistently negative workup is perplexing.

Zhang et al. suggested performing a low-dose dexamethasone suppression test in individuals presenting with PA, prior to adrenal vein sampling (AVS) and surgery due to the high prevalence of Cushing’s syndrome in patients with PA [8]. A positive test result can lead to a straightforward diagnosis; however, in this rare case where the patient had severe negative tests, it can present as a challenge in diagnosis and treatment.

## Conclusions

The presence of PA and concurrent Cushing’s syndrome can present as a diagnostic challenge. It is recommended to follow up on the signs of Cushing's syndrome with preliminary tests and to presume its absence if two concordant tests are negative. Our patient, however, was an exceptional case.

This case highlighted the importance of maintaining a high index of suspicion for patients presenting with several signs and symptoms of the disease and a negative workup. More attention should be paid to the patient's history, and a thorough physical examination should be conducted. In those with an uncertain diagnosis, adrenal venous sampling can provide a clearer picture and lead to a more accurate understanding of the case.
